# The proteolytic landscape of cells exposed to non-lethal stresses is shaped by executioner caspases

**DOI:** 10.1038/s41420-021-00539-4

**Published:** 2021-06-19

**Authors:** María del Carmen Conde-Rubio, Roman Mylonas, Christian Widmann

**Affiliations:** 1 Department of Biomedical Sciences, University of Lausanne, Bugnon 7, Lausanne, Switzerland; 2 Protein Analysis Facility, University of Lausanne, Génopode, Lausanne, Switzerland; 3 SIB Swiss Institute of Bioinformatics, Amphipole, Lausanne, Switzerland

**Keywords:** Apoptosis, Protein quality control

## Abstract

Cells are in constant adaptation to environmental changes to insure their proper functioning. When exposed to stresses, cells activate specific pathways to elicit adaptive modifications. Those changes can be mediated by selective modulation of gene and protein expression as well as by post-translational modifications, such as phosphorylation and proteolytic processing. Protein cleavage, as a controlled and limited post-translational modification, is involved in diverse physiological processes such as the maintenance of protein homeostasis, activation of repair pathways, apoptosis and the regulation of proliferation. Here we assessed by quantitative proteomics the proteolytic landscape in two cell lines subjected to low cisplatin concentrations used as a mild non-lethal stress paradigm. This landscape was compared to the one obtained in the same cells stimulated with cisplatin concentrations inducing apoptosis. These analyses were performed in wild-type cells and in cells lacking the two main executioner caspases: caspase-3 and caspase-7. Ninety-two proteins were found to be cleaved at one or a few sites (discrete cleavage) in low stress conditions compared to four hundred and fifty-three in apoptotic cells. Many of the cleaved proteins in stressed cells were also found to be cleaved in apoptotic conditions. As expected, ~90% of the cleavage events were dependent on caspase-3/caspase-7 in apoptotic cells. Strikingly, upon exposure to non-lethal stresses, no discrete cleavage was detected in cells lacking caspase-3 and caspase-7. This indicates that the proteolytic landscape in stressed viable cells fully depends on the activity of executioner caspases. These results suggest that the so-called executioner caspases fulfill important stress adaptive responses distinct from their role in apoptosis. Mass spectrometry data are available via ProteomeXchange with identifier PXD023488.

## Introduction

Cells are permanently adapting to environmental alterations. They must gauge the extent of stresses they are exposed to in order to determine when survival and repair pathways need to be activated or when suicide programs have to be triggered when the sustained damage is too extensive. These adaptive modifications can be mediated by the selective modulation of gene and protein expression as well as by post-translational modifications (PTMs) [1, 2], such as phosphorylation and proteolytic processing [3]. Protein cleavage, when controlled and limited to specific proteins, is involved in diverse physiological processes such as the maintenance of protein homeostasis and the regulation of growth. Additionally, proteolysis plays critical functions in apoptosis and in the activation and regulation of different stress response pathways [4].

Apoptosis, leading to controlled elimination of damaged cells, is orchestrated by members of the caspase (cysteine aspartate specific proteases) family of cysteine proteases. Caspase-mediated proteolysis also participates in diverse processes such as inflammation regulation [5], development [6], and even cell protective responses [7].

During apoptosis, upstream caspases (caspase-8, −9) drive the cleavage and the activation of the down-stream caspases (caspase-3, −6 and −7). The latter are also called executioner caspases and mediate the cleavage of hundreds of proteins leading to the demise of apoptotic cells [8]. Caspase-3 (CASP3) and caspase-7 (CASP7) are considered the main apoptosis executioners because, contrary to caspase-6, their activation is apparently sufficient for cells to trigger apoptosis [9]. CASP3 and CASP7 recognize similar sequences and have many substrates in common [10] but they are not entirely functionally redundant, CASP3 being apparently more promiscuous than CASP7 and playing a more predominant role in the demolition phase of apoptosis [11].

CASP3 can also mediate physiological functions other than apoptosis [12]. It is involved in the terminal differentiation of human and murine erythroblasts [13], in embryonic stem cell differentiation [14] and in the negative regulation of B-cell cycling [15]. Paradoxically, in mild stress conditions, CASP3, rather than favoring cell death, activates cell survival signaling [16]. It must be borne in mind, that in these situation, CASP3 activity levels are distinctively lower than those observed in apoptotic cells [17].

In addition to caspases, other proteases, such as calpains, cathepsins, the ubiquitin-proteasome system and granzymes, are activated during apoptosis [18, 19]. Their role in apoptosis is however still incompletely understood.

The function of the cleavage of the >700 reported caspase substrates (see the CASBAH website [20]) has been thoroughly characterized in a few cases only. Optimally, this requires generation of knock-in cells or animals having their wild-type caspase substrates replaced with cleavage-resistant mutants and assessment of the physiological consequences of these replacements. Such approach has been performed for the Rb protein [21, 22], p120 RasGAP [16, 23], β-amyloid precursor protein, and PARP1 (ref. 24). What is the set of caspase substrates that need to be cleaved for apoptosis to proceed normally and what are the caspase substrates that are cleaved but that play no significant function in apoptosis—substrates often dubbed “innocent bystanders”—are questions that still need to be answered.

Proteomics approaches have the power to identify a large number of severed proteins simultaneously and can provide some information about the nature of the cleavage sites. However, these approaches tend to detect only the most abundant proteins. Moreover, these techniques may be sensitive to other types of PTMs such as ubiquitination, acetylation and phosphorylation [25]. It is important to note that mass spectrometry analyses performed so far to detect cleaved proteins in apoptotic cells did not provide information on the exact identity of the protease(s) that are involved in the observed cleavages [26–31].

In contrast to the interest for proteins cleaved during apoptosis, very little has been done on the extent and nature of protein cleavage in cells subjected to non-lethal forms of stresses. In this study, we provide the global proteolytic landscapes in two different human cancer cell lines (the osteosarcoma U2OS and the colorectal carcinoma HCT116) in both mild stress and apoptotic conditions. Using CASP3/CASP7 double knock-out (DKO) HCT116 cells, we determined the contribution of these caspases in the establishment of these proteolytic landscapes. Our results indicate that CASP3 and CASP7 are required for the cleavage of all proteins that we detected to be cleaved upon mild stress exposure, while in apoptosis the cleavage of about 20% of the cleaved proteins depended on other proteases.

We also generated an interactive database (The Cleavage Profiling database, https://cleaved-apoptosis.unil.ch) listing the proteins experiencing PTMs after the induction of apoptosis and upon mild stress exposure. This database provides information on primary and secondary protein structures, types of PTMs, putative cleavage site(s), and function of the cleavage when known.

## Methods

### Antibodies

The anti-PARP1 (ref. no.: 9542 S, lot no. 14), anti-caspase-7 (ref. no.: 9492 S, lot no. 6) and anti-caspase-3 (ref. no.: 9662, lot no. 18) antibodies were from Cell Signaling Technology. Antibodies against Telo2 and Secernin-1 are from Abcam (ref. no. ab122722, lot no. GR3211414-3) and GeneTex (ref. no.: GTX85172, lot no. 821805738), respectively. The secondary rabbit antibody was purchased from Thermo Fisher Scientific (goat anti-Rabbit, Alexa Fluor 680, catalog # A-21109, RRID AB_2535758, lot no. 1584296). The anti-GFP antibody was purchased from Roche (ref no. 11814460001, lot no. 11063100) and the anti-Flag antibody was obtained from Merck (ref no., F1804–50UG, lot no. SLBK1346V). Additional antibodies are described in Table 2.

### Cell counting

Following the indicated treatments, cells were washed with 10 ml of 1X phosphate-buffered saline (PBS, 0.116 M NaCl, 10.56 mM Na_2_HPO_4_, 2.94 mM KH_2_PO_4_, pH adjusted to 7.1–7.3), detached using 1 ml of trypsin-EDTA (0.05%) (Thermo Fisher Scientific, ref. n°. 25300054, lot n° 1854719) and resuspended in 5 ml of DMEM medium. Then 50 μl of each cell suspension were stained at room temperature for 5 min with 50 μl of PBS, 0.4% trypan blue (Gibco, ref. no. 15250061, lot no. 1903978). Dead (trypan blue-positive) and live (trypan blue-negative) cells were counted in a Neubauer chamber (Blau Brand, ref. no. 718605, lot no. 03H) using a Nikon Eclipse TS100 microscope. Four measurements were performed for each determination.

### Cell lines

U2OS human osteosarcoma, HCT116 colon carcinoma cell lines and HEK 293T human embryonic kidney cells were purchased to the American Type Culture Collection (ATCC). NB1 cells carrying the LEGO-iG2.lti plasmid (#807) (ref. 32) were generated as described earlier [33]. All cell lines were cultured in Dulbecco’s modified Eagle medium (DMEM; Gibco, ref. no. 61965) supplemented with 10% heat-inactivated fetal bovine serum (FBS; Invitrogen, ref. no. 10270-106, lot no. 42G8468K) at 37 °C in 5% CO_2_.

### Chemicals

Z-VAD-FMK was purchased from Enzo Life Sciences (ref. no. BML-P416-0001). Oligonucleotides were purchased from Microsynth, Balgach, Switzerland. Cisplatin (ref. no. P4394-25MG) was acquired from Sigma-Aldrich. Propidium iodide (PI, Sigma-Aldrich, #81845) was stored at −20 °C after being aliquoted (400 μg/ml) in water.

### Colony formation assays

After incubation with the treatments mentioned in the figures, cells were trypsinized and counted as described above. The cells were then seeded in 100 mm × 20 mm plates (Corning, ref. no. 430167) at a density of 500 cells/ well. Ten days later, the plates were washed with 10 ml PBS, dried upside down for 1 h, fixed with 100% ethanol (Reactolab, ref. no. 15058) for 1 h, and finally dried for an additional hour. Thereupon, cells were stained with 10 ml Giemsa solution (AppliChem, ref. no. A0885, lot no. 5×001792) for 30 min. The Giemsa solution was removed and the background staining was eliminated by gently immersing the plates once or twice (until background removal) in an ice bucket (Bel-Art™, ref. no. M18848-4002) filled with 3 liters of water. Finally, plates were dried and colonies (clusters of at least 2 cells) and single cells were counted using a Nikon Eclipse TS100 microscope.

### CRISPR/Cas9-mediated CASP3 and CASP7 gene disruptions

The generation of the CASP3/CASP7 DKO HCT116 cell was done using a stepwise approach. CASP3 knock-out (KO) HCT116 cells were first generated and then used to knock-out CASP7. Knocking-out CASP3 was achieved following transient transfection of sgRNA[hCaspase-3] [2]. ltiCRISPRv2 in HCT116. Transient transfection was used to avoid permanent expression of Cas9 in cells. For the transfection, X-tremeGENE 9 DNA Transfection Reagent (Sigma-Aldrich, ref. no.: 06 365 809 001) was used according to the manufacturer’s instructions. Briefly, 120,000 cells were plated in 6 well plates (Corning, ref. no. 3516) containing 2 ml of DMEM (Gibco, ref. no. 61965). The following day, 100 μl Opti-MEM (Thermo Fisher Scientific, 11058021) were added in a sterile tube, together with the siRNA oligonucleotides. Then, 2.5 μl of X-tremeGENE 9 DNA Transfection Reagent were added and the content was mixed. After 15 min of incubation at room temperature, the mixture was added dropwise in the wells containing the cells. Cells that were not transfected were eliminated by the addition, 24 h post-infection, of 3 μg/ml of puromycin for 72 h. Clone isolation was performed by limiting dilution in 96-wells plates. Twelve U2OS clones and nine HCT116 clones were obtained through this procedure. The presence or the absence of CASP3 and Cas9 was assessed by western blotting (Supplementary Fig. S6). The sequences in the vicinity of the region targeted by the CRISPR/Cas9 system were determined in the clones lacking CASP3 and Cas9, following PCR amplification of the regions of interest, using the primers listed in Supplementary Table 5, cloning the PCR fragments into pCR®2.1 vector using TA cloning (Life technologies, ref n°K202020), according to manufacturer’s instructions, and sequencing of the inserts of these plasmids.

Using a validated CASP3 KO cell clone, the same procedure as above was used to disrupt the CASP7 alleles. The functionality of these CASP3/CASP7 DKO HCT116 cells was performed analyzing their ability to cleave PARP-1. It was abolished (Supplementary Fig. S6).

### Flow cytometry

Cells (120,000 per well) were seeded in 6 well plates containing 2 ml of DMEM supplemented with 10% heat-inactivated FBS. The following day, cells were treated as indicated in the figures. Cells were pelleted, washed with cold PBS and resuspended in 500 µl cold PBS containing 3 μg/ml of propidium iodide to stain dead cells. Samples were transferred to cytometer tubes (Sarstedt, #55.1759) and analyzed by flow cytometry (FC-500, Beckman Coulter) as described [34]. Data were analyzed with the Kaluza (Version 1.2) software (Beckman Coulter). Gating parameters are described in Supplementary Table 2.

### Plasmids

Single guide RNAs (sgRNAs) targeting the proteins of interest (CASP3 and CASP7) were selected from a published library made available by the Feng Zhang’s laboratory [35]. Plasmids sgRNA[hCaspase-3] [2].ltiCRISPRv2 (#1010) and sgRNA[hCaspase-7] [5].ltiCRISPRv2 (#1011) targeting CASP3 and CASP7, respectively, were created according to the protocol described in ref. [36] using the oligonucleotides listed in Supplementary Table 1.

Plasmid psPAX2 (#842) (Addgene, ref. no. 12260) is a second-generation lentiviral packaging vector and pMD2.G plasmid (#554, Addgene, ref. no. 12259) encodes the lentivirus envelope. VC3AI.lti (#852), a lentiviral vector encoding a Venus-based caspase-3-like sensor, was a gift from Binghui Li (Laboratory of Cancer Cell Biology, Key, Tianjin Medical University Cancer Institute and Hospital, Tianjin, 300060, China). The lentiviral lentiCRISPR v2 (#868) was a gift from Feng Zhang (Addgene plasmid # 52961; http://n2t.net/addgene:52961; RRID:Addgene_52961) (ref. 37).

### Slice-SILAC methodology

### A. SILAC labeling

VC3AI HCT116, CASP3/ CASP7DKO VC3AI HCT116 and VC3AI U2OS cells were cultured in SILAC-compatible DMEM medium (Thermo Fisher Scientific, ref. n°89985) supplemented with either “light” (L) or “heavy” (H) stable isotope-labeled L-arginine and 150 mg/l of L-lysine to obtain a final concentration of 50 mg/l and 150 mg/l respectively. These amino acids were purchased from Sigma: proline 0 (ref. n° P-5607), arginine 0^1^ (ref. no. A-8094), lysine 0 (ref. no. L5501), lysine+8 (ref. no. CNLM-291-H0.1), arginine +10 (ref. no. CNLM-539-H0.1). The media were supplemented with 10% dialyzed fetal bovine serum (Sigma-Aldrich, F0392, lot no. 16M280-A) and L-proline 0 (final concentration of 200 mg/l). The addition of L-proline 0 is to prevent the conversion of arginine to proline [38]. Cells went through 6 cell cycle of divisions before assessing, by mass spectrometry, the levels of labeled amino acids in actin. At this point, labeling was higher than 95% and no proline conversion was detected. Two more cell divisions were allowed to proceed to enrich the labeling further. The cells were then used as indicated in the next sections. H-labeled cell extracts were used as reference while all treated samples were L-labeled.

### B. Induction of stress and protein extraction and quantification

Cells were seeded at a density of 120’000 cells/well in 6 well plates (Corning, ref. no. 3516). The following day, cells were treated or not with 4 µM cisplatin (non-lethal stress condition) or 32 µM (apoptotic condition) for 48 h. Cells were then lysed in FASP buffer (4% of sodium dodecyl sulfate [SDS], 0.1 M of DL-Dithiothreitol (DTT) and 100 mM of Tris pH 7.5). The lysates were heated at 95 °C for 5 min and sonicated for 3 cycles of 5 s followed by centrifugation at 16,000 g for 10 min to pellet nuclei and cellular debris. Then, the soluble cell extracts were subjected to sodium dodecyl sulfate-polyacrylamide gel electrophoresis (SDS-PAGE). Proteins present in the gel were detected with Coomassie staining. Gels were imaged and protein concentrations were determined by densitometry scanning of whole lanes of SDS/PAGE gels [39].

### C. Protein separation and mass spectrometry

Lysates of light and heavy amino acid-labeled cells were mixed at a quantitative ratio of 1:1. A total of 120 μg of protein from mixed lysates was migrated on a pre-cast 4–12% Novex NuPAGE Bis-Tris SDS Mini Gel (Invitrogen). Entire lanes were cut into 46 identical gel slices using GridCutter (Gelcompany). In-gel proteolytic cleavage with sequencing grade trypsin (Promega) was performed as described [40]. The peptides from the digestion were dried and redissolved in 0.05% trifluoroacetic acid, 2% acetonitrile for analysis with liquid chromatography coupled with tandem mass spectrometry. Samples were injected on a Q-Exactive Plus mass spectrometer interfaced via a nano EASY-Spray source to an Ultimate 3000 RSLCnano HPLC system (Thermo Scientific). After loading onto a trapping microcolumn Acclaim PepMap100 C18 (20 mm×100 μm ID, 5 μm, Thermo Scientific), peptides were separated on a reversed-phase Easy Spray C18 column (50 cm×75 µm ID, 2 µm, 100 Å, Thermo Scientific). A 4-76% acetonitrile gradient in 0.1% formic acid (total time 65 min) was used for the separation with a flow of 250 nl/minute. Full MS survey scans were performed at 70,000 resolution. In data-dependent acquisition controlled by Xcalibur 3.0.63 software (Thermo Scientific), the 10 most intense multiply charged precursor ions detected in the full MS survey scan were selected for higher energy collision-induced dissociation (HCD, normalized collision energy NCE = 27%) and analyzed in the orbitrap at 17’500 resolution. The window for precursor isolation was of 1.5 m/z units around the precursor and selected fragments were excluded for 60 s from further analysis.

### D. MS data analysis

MS data were analyzed and quantified using the MaxQuant software (version 1.5.3.3) (ref. 41), and the Andromeda search engine set to mine the human proteome in the UniProt database (UniProt proteome ID: UP000005640, version of July 2017, number of sequences: 20,987). Trypsin was defined in MaxQuant as the enzyme used to digest the proteins (selected setting: “cleavage after lysine and arginine”). As complete protein digestion often does not occur [42], peptides with up to two uncut arginine/lysine (i.e., 2 missed cleavages) were taken into account. As proteins were subjected to DTT to reduce their disulfide bonds, the carbamidomethylation of cysteine was specified as a fixed modification, while N-terminal acetylation of protein and oxidation of methionine were specified as variable modifications. Protein identifications were filtered at 1% false discovery rate established against a reversed database, according to default MaxQuant parameters. Sets of protein sequences which could not be discriminated based on identified peptides were listed together as protein groups. MaxQuant analysis used an experimental design file in which every dataset corresponding to a gel slice was quantified separately as a distinct experiment, numbered as the gel slice it corresponded to. According to this previously described method [43], results from MaxQuant were then filtered and processed further with custom-built R scripts. The main MaxQuant result (proteinGroups.txt) was filtered to identify proteins present in at least two distinct gel slices with different SILAC ratios. A molecular weight (MW) scale of the gel slices was built based on a polynomial regression fit of protein theoretical weights. Proteins with an H/L ratio below 4 and probable contaminant proteins, observed in more than 30% of the slices, were not used for the regression fit. Just proteins present in at least two gel slices, with a minimum H/L ratio of 2 in every slice and present in at least one slice with a log_2_(H/L) value higher and or lower to 0.5, were considered and displayed in the raw data.

To assign proteins as post-translationally modified, they have to have, for a given cell line and a given stress exposure, the same log_2_(H/L) value pattern in at least two experiments. Further visual classification according to their exhibited PTM (discrete fragmentation, ubiquitination, tagging) was then performe. Supplementary Table 3 indicates the number of experiments performed for each condition and cell line.

### Popviz interface

PopViz is a multitier web application with a backend written in PHP (v5.5) (ref. 44) using the Slim framework (v3.9) (ref. 45). The frontend is based on ReactJS (v16.0) (ref. 46) and most of the visualization is done using the D3.js library (v4.11) (ref. 47). MongoDB (v3.6) (ref. 48) is used to store and query the data. The database is populated with JSON objects that are created by processing MaxQuant result files (proteinGroups.txt and peptides.txt) using the statistical programming language R (v3.6) (ref. 49).

The first step in data processing was to remove from the proteinGroups.txt file entries marked as “Potential contaminant”. Furthermore, entries matching an additional list of known contaminants were removed using an R package provided at https://github.com/UNIL-PAF/PAFcontaminants. To determine the relationship between migration distance in the SLICE-SILAC gel and the molecular masses of the migrated proteins, a third-degree polynomial function was fitted to a plot of theoretical protein masses against its slice position in the gel. Normalization of the H/L ratios was done using a zero-median normalization for each slice. Slice-to-mass fitting and normalization were done for each dataset separately.

The Popviz interface also provides information on cleavage sites derived from a database [50] generated and hosted by the Melbourne University (https://sunflower.kuicr.kyoto-u.ac.jp/~sjn/Cascleave/database.html). This database uses a collection of different databases on protein cleavage, such as The CASBAH [20], MEROPS [51] and CASVM [52]. It contains 370 caspase substrate sequences and 562 cleavage sites. After filtering, slice-to-mass fitting, normalization and adding the cleavage information, a separate JSON entry for each protein group was created. Only proteins listed in “Majority protein IDs” are considered as valid proteins. For every protein the corresponding peptide information was parsed and added from the peptides.txt file.

The source code described here, used to create PopViz, is available on GitHub [53] and archived in Zenodo [54] (PopViz backend: DOI 10.5281/zenodo.4335268 and PopViz frontend: DOI 10.5281/zenodo.4337164).

### Secernin-1 and Telo2 siRNAs

To validate the antibodies against Secernin-1 and Telo2, these proteins were downregulated with specific siRNAs (Supplementary Table 4). They were designed using the bioinformatics tool “siRNA at Whitehead” (http://sirna.wi.mit.edu/home.php), considering the Consensus CDS sequences CCDS5422.1 and CCDS32363.1, for Secernin-1 and Telo2 respectively. We verified that these siRNAs targeted all the protein isoforms using SliceCenter [55].

### siRNA transfection

Cells were seeded in 2 ml of culture medium in 6-well plates at a 150,000 cells/well density. Twenty-four hours later, cells were transfected with 20 nM siRNAs for a period of 48 h using the X-tremeGENE 9 DNA Transfection Reagent (Sigma-Aldrich, ref. no.: 06 365 809 001) as described in section “*CRISPR/Cas9-mediated CASP3 and CASP7 gene disruptions*”.

### VC3AI HCT116, VC3AI U2OS and CASP3/CASP7 DKO VC3AI HCT116 cell line generation

Lentiviruses encoding the VC3AI executioner caspase sensor [36] were produced as described [56] with the following modification: pMD.G (#218) and pCMVΔR8.91 (#219) were replaced by pMD2.G and psPAX2 (#842) respectively, and the lentiviral vector used was VC3AI.lti (#852).

HEK293T cells were co-transfected with 12.5 µg of pMD2.G (#554), 37.5 µg of psPAX2 and 50 µg of VC3AI.lti.cm3.dna using the calcium phosphate method [57]. The supernatant was collected 48 post-transfection and centrifuged at 16,000 g for 5 min to harvest the virus. U2OS, HCT116 and CASP3/CASP7 DKO HCT116 cell lines were infected with the viruses and selected with 3 µg/ml of puromycin (Thermo Fisher Scientific, A1113802, lot no. 1970741) for 48 h. Clone isolation was performed for VC3AI HCT116 and VC3AI U2OS by limiting dilution in 96-well tissue culture plates in the absence of puromycin. The VC3AI HCT116 and VC3AI U2OS clone validations and the whole cell population CASP3/CASP7 DKO VC3AI HCT116 confirmation was carried out by western blotting using an anti-GFP antibody as previously described [58], to detect the Venus-derived CASP3/7 activity sensor encoded by the VC3AI.lti plasmid (Supplementary Fig. 1).

### Western blot

Following the treatments described in the figures, cells were trypsinized as described in the “*Cell counting”* section, washed with 10 ml PBS, pelleted and lysed for 15 min in 50 µl NP40 buffer (50 mM Tris-HCl, 150 mM NaCl, 1% NP40) containing phosphatase (PhosSTOP™, Sigma-Aldrich, ref. no. 4906837001) and protease inhibitors (cOmplete™ Protease Inhibitor Cocktail, Sigma-Aldrich, ref no. 04693116001). Then, samples were centrifuged at 15,000 x g for 10 min at 4 °C and the supernatants were collected. Protein concentration was measured using the Bradford procedure [59] and 40 µg of each sample were then diluted in Laemmli 5x sample buffer (312 mM Tris-HCl pH 6.8, 10% SDS, 50% glycerol, 25% β-mercaptoethanol, 0.1% bromophenol blue) and boiled at 95 °C for 10 min before being loaded on 10% (for PARP1 detection) or 12% (for caspase-3 and caspase-7 detection) SDS-PAGE gels. Proteins were transferred to a 0.45 µm nitrocellulose membranes (Biorad; ref. no. 1620115) and then membranes were blocked for one hour at room temperature in 5% non-fat milk diluted in TBST [130 mM NaCl, 20 mM Tris-HCl (pH 7.2), 18 mM HCl, 0.1% Tween-20] and then they were incubated overnight at 4 °C with gentle shaking with primary antibodies (1:1000 dilution in TBST, 5% non-fat milk). Membranes were then washed three times for 20 min with TBST and then incubated for one hour at room temperature with the secondary antibody (1:5000 dilution in TBST, 5% non-fat milk). After three additional washing with TBST, western blots were imaged using the Odyssey Infrared Imager (LI-COR, 9120).

## Results

### Defining the stress conditions leading to either cell survival or apoptosis

We have shown earlier that caspase-3, when mildly activated by low stresses in non-dying cells, cleaves the p120RasGAP protein into an N-terminal fragment with potent anti-apoptotic activity [7, 60]. In the present study, we wanted to determine if other proteins are cut in conditions where caspase-3 is activated in cells that are not undergoing apoptosis. We used the U2OS osteosarcoma and HCT116 colon carcinoma as the cell line models and a genotoxin, cisplatin, as a generic apoptotic stress inducer [61].

The experimental condition leading to a non-lethal form of stress was defined as the concentration of cisplatin inducing detectable CASP3-like activity but that did not negatively impact on cell survival and that did not lead to marked cleavage of PARP-1 [62], a classical CASP3 substrate cleaved during apoptosis [63]. The apoptotic condition was defined by a cisplatin concentration that induced elevated CASP3-like activity, pronounced PARP-1 cleavage and cell death.

CASP3-like activity was measured in HCT116 and U2OS clones expressing the sensitive fluorescent-based VC3AI biosensor (Supplementary Figure S1A; these clones are called VC3AI HCT116 and VC3AI U2OS cells, respectively) that enables CASP3-like activity visualization and quantification in single cells [29]. This sensor is not fluorescent unless cleaved at a specific CASP3 recognition site [29]. These cells were treated with increasing concentrations of cisplatin for 24, 48, and 72 h (Supplemental Figs. S2 and S3). An exposure time of 48 h was deemed suited to for the differentiation of CASP3-like activities between cells treated with low and high cisplatin concentrations.

**Fig. 1:**
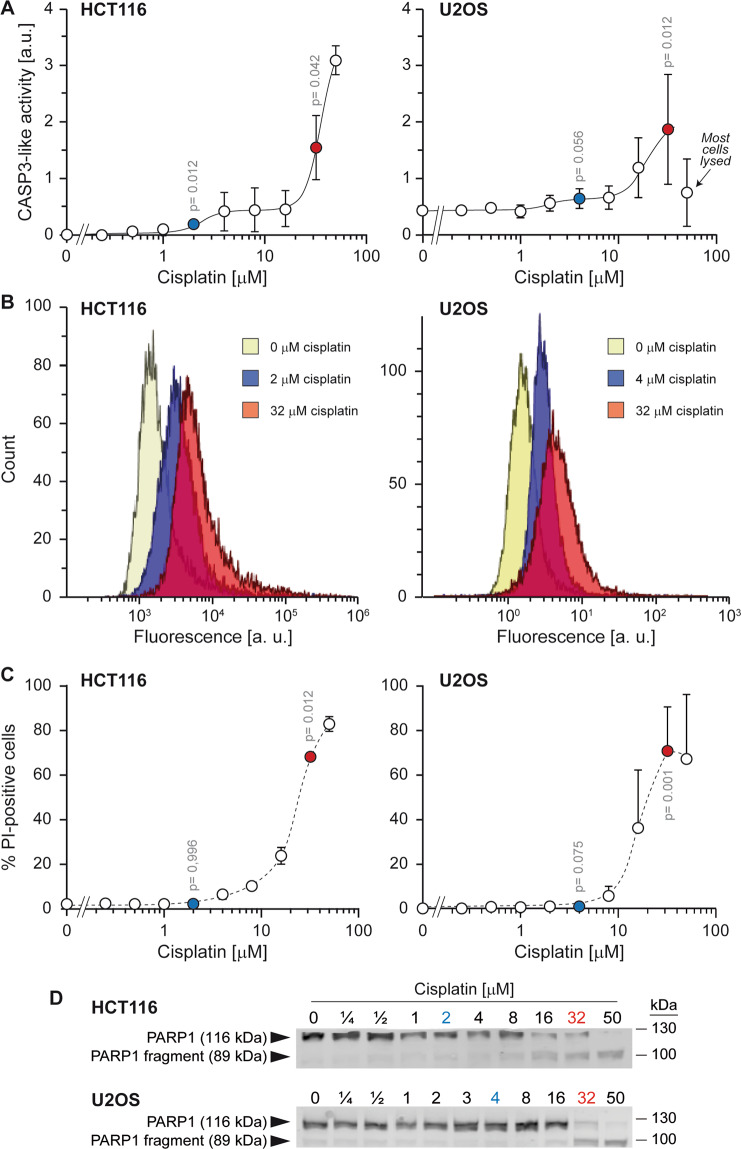
Monitoring CASP3-like activity, cell death and apoptosis in HCT116 and U2OS cell lines upon treatment with increasing cisplatin concentrations. HCT116 and U2OS cells stably expressing the VC3AI CASP3-like activity sensor (120,000 cells seeded in 2 ml of DMEM supplemented with 10% heat-inactivated FBS in wells of 6-well plates) were treated for 48 h with the indicated cisplatin concentrations. **A**–**B** Fluorescence of the VC3AI sensor was recorded by flow cytometry. The data in panel **A** correspond to the mean (autofluorescence of the cells subtracted; see Figure S4) ±standard deviation (SD) of four independent experiments. Statistical analyses were performed using paired *t*-test. Panel **B** shows the flow cytometry profiles of cells exposed to the cisplatin concentrations highlighted in blue and red in panel **A**. **C** Cell death was recorded by flow cytometry following PI staining. The data correspond to the mean ± SD of four independent experiments. Statistical analyses were performed using paired *t* test. **D** PARP1 cleavage was assessed by western blotting.

**Fig. 2:**
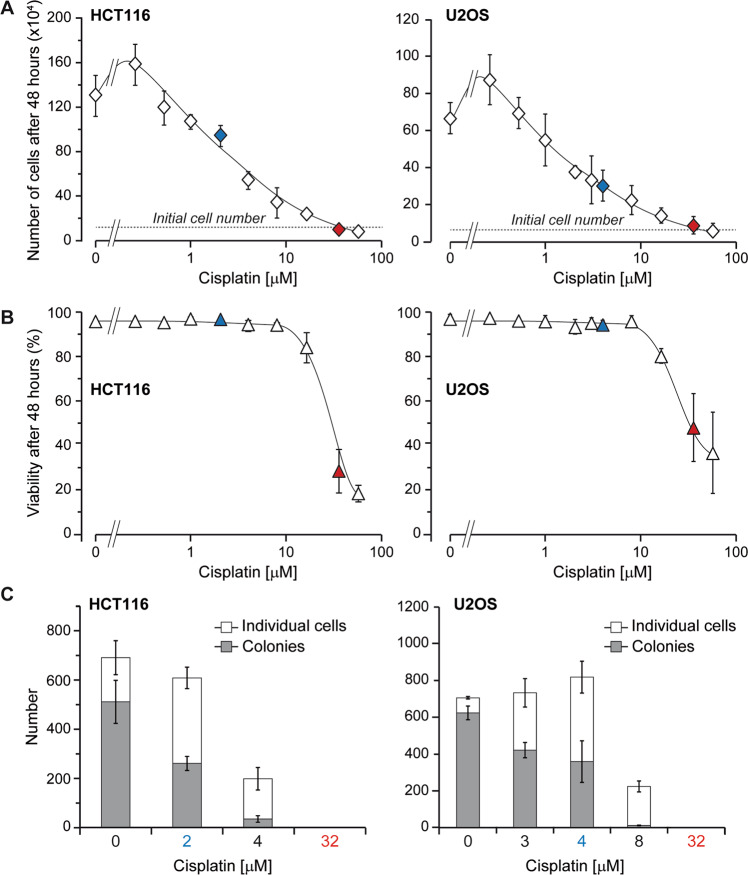
Viability and proliferative capacity of cells exposed to increasing cisplatin concentrations. HCT116 and U2OS WT cells (120,000 cells seeded in wells of 6-well plates) were treated for 48 h with the indicated cisplatin concentrations. **A**–**B** Viability (panel **A**) and cell number (panel **B**) were determined through trypan blue staining. The results correspond to the mean ± 95% confidence interval (CI) of three independent experiments. **C** Following the 48 h incubation with the indicated cisplatin concentrations, cells were trypsinized and five hundred of them were plated in 10-cm Petri dishes and grown for 10 days in culture medium without cisplatin. The number of colonies (cluster of 2 or more cells) and isolated cells (single cell “colonies”) in the plates was then recorded. Results correspond to the mean ± 95% CI of two independent experiments. Conditions highlighted in blue and red correspond to cisplatin concentrations that activate low CASP3-like activity in live cells and high CASP3-like activity in apoptotic cells, respectively (see Fig. 1).

The autofluorescence of wild-type HCT116 and U2OS cells increased as a function of cisplatin exposure (Supplementary Fig. S4). Therefore, in subsequent experiments assessing the CASP3-like activity in clones expressing the VC3AI sensor, we subtracted the autofluorescence recorded at given cisplatin concentrations. Figure 1A shows that CASP3-like activity in response to increasing cisplatin concentrations displayed a biphasic response of low and strong CASP-3 activities. Incubation with 2 µM and 4 µM of cisplatin for 48 h in HCT116 and U2OS cells, respectively, generated a discrete and homogenous caspase activity, that was distinctively lower than the caspase activity generated when apoptosis was induced with higher (32 µM) cisplatin concentrations (Fig. 1B). The presence of only one peak in the flow cytometry profile of VC3AI-expressing cells exposed to low cisplatin concentrations indicates that CASP3-like proteases are activated to similar extent in all cells. In conditions of low CASP3-like activities induced by 2 µM and 4 µM of cisplatin in HCT116 and U2OS cells, respectively, cells did not undergo apoptosis (Fig. 1C) and PARP1 was not cleaved into the characteristic 89 kDa apoptotic fragment (Fig. 1D). In these conditions moreover, cells were still able to proliferate and form colonies, albeit to a lower extent compared to cells that were not exposed to cisplatin (Fig. 2). Hence, we could define situations where mild activation of CASP3-like proteases does not compromise cell survival and only partially impairs their proliferation.

### PTMs in mild stress and apoptosis

**Fig. 3:**
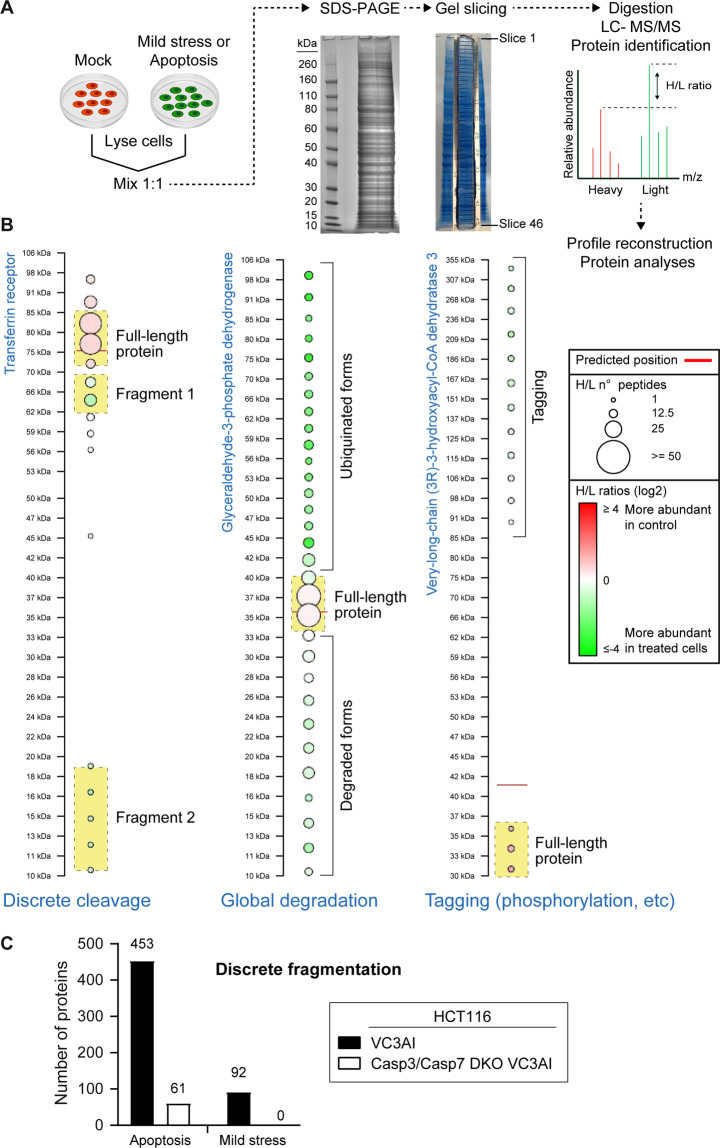
PTMs in mild stress and apoptosis. **A** Scheme depicting the SLICE-SILAC methodology. Cells labeled with normal (light) or heavy amino acid isotopes (red and green cells, respectively) are exposed to two different experimental conditions (in our case exposed or not to a given cisplatin concentration). The two populations are then mixed and loaded on an SDS-PAGE gel. Upon completion of electrophoresis, the gel is cut into ~50 slices that are individually subjected to trypsin digestion and analyzed by liquid chromatography with tandem mass spectrometry (LC–MS/MS). Because of the differential isotope labeling, it is possible to determine from which experimental condition a mass spectrometry-identified protein is coming from. Identification of a protein in a gel slice that does not correspond to its MW indicates that the protein has been subjected to PTMs. **B** Three examples of PTMs detected by the SLICE-SILAC methodology. **C** Number of proteins detected by the SLICE-SILAC procedure as cleaved under non-lethal stress and during apoptosis in VC3AI HCT116 and CASP3/CASP7 DKO VC3AI HCT116 cells.

We selected 2 µM and 4 µM cisplatin exposure for 48 h in VC3AI HCT116 and VC3AI U2OS cells, respectively, as the experimental conditions to investigate the proteolytic landscape of proteins in cells subjected to non-lethal stress. An exposure to 32 µM cisplatin for 48 h defined the apoptotic condition. Identification of pattern consistent with different PTMs, including cleavage events, was performed by SLICE-SILAC (Fig. 3A). Examples of patterns that could be ascribed to specific PTMs are shown in Fig. 3B. The appearance of discrete fragments with a concomitant decrease in the levels of the full-length protein is indicative of protein cleavage by proteases at specific sites (left example in Fig. 3B). Formation of a ladder of protein species above the full-length protein is indicative of ubiquitination. This can be accompanied (middle example in Fig. 3B) or not by the presence of a ladder of fragments below the full-length protein, the latter case corresponding to global, proteasome-mediated, degradation. The appearance of discrete specie(s) above the full-length protein is indicative of covalent linkage of structures to the protein following events such as phosphorylation, acylation, glycosylation, etc. This type of pattern is coined “tagging” here. When it was not possible to assign a PTM to one of the examples shown in Fig. 3B, the PTM was classified as “other”. Undefined PTMs may originate from the combination of several PTMs, from the addition of structures such as palmitoyl groups that hamper peptide identification by mass spectrometry [43], or even from the stress-related appearance of splice variants presenting different gel migration patterns. PTMs occurring on low abundance proteins are often inaccurate due to the poor identification of scarce proteins by mass spectrometry [64]. In this context, we note that the proteins found here to be post-translationally modified are generally rather abundant proteins (Supplementary Figure S5). This is also due to the fact that PTMs happens at a sub-stoichiometric level [65].

A total of 1006 proteins were found to have their gel migration pattern modified upon apoptosis induction (Table 1 and Excel S1). Most of these changes were consistent with proteolysis (Table 1). Upon induction of non-lethal stress, 397 proteins with distinct migration pattern were detected (Table 1 and Excel S2). Interestingly, discrete cleavage of proteins was not the predominant type of PTMs detected upon mild stress induction as the different types of pattern consistent with different PTMs occurred with relatively similar frequencies in this condition (Table 1). We also detected that in both conditions, several cleaved proteins exhibited additionally other kinds of modification (Excel S3). Excel S4 lists the proteins that were cleaved in VC3AI HCT116, VC3AI U2OS, or both cell lines, exposed to either non-lethal cisplatin concentrations or to apoptosis-inducing cisplatin concentrations.

**Table 1:** Number of detected proteins with specific PTMs pattern induced by mild stress and apoptosis.

PTM pattern	Mild stress				Apoptosis			
	HCT116	U2OS	Both	Total	HCT116	U2OS	Both	Total
Discrete fragmentation	22	85	15	92	420	130	99	453
Ubiquitination	22	86	21	87	190	62	58	194
Tagging	42	77	18	101	241	55	41	255
Degradation	20	86	9	97	248	62	60	250
Not well defined	8	63	8	79	66	0	2	67

This table lists the number of proteins detected by SLICE-SILAC that bear the indicated PTMs found in VC3AI HCT116 and VC3AI U2OS incubated with low or high cisplatin concentrations. Some proteins presented more than one type of PTMs.

About 70% of the proteins that we found to be cleaved during apoptosis in VC3AI HCT116 and VC3AI U2OS cells were already described as protease substrates in the DegraBase [66], the largest proteolysis database reporting the cleavage of proteins in apoptosis as well as under normal conditions. The remaining 30% (127 proteins) are therefore newly identified protease substrates in cells undergoing cell death (Excel S5). All the proteins cleaved under mild stress conditions were also found to be cleaved during apoptosis (Excel S4) with the notable exception of Cullin-5 and CLIP-associating protein 1. The cleavage pattern of some of these proteins was different in cells exposed to non-lethal stress and in cells undergoing apoptosis (Excel S6).

We built a web interface, called the ClePro (Cleavage Profiling) database (https://cleaved-apoptosis.unil.ch/), which reports information about the detected PTMs, in VC3AI HCT116 and VC3AI U2OS cell lines, upon non-lethal stress exposure and apoptosis. This resource provides general information on the detected proteins, the type of PTMs they have experienced, and previous information on the cleavage of these proteins when known. Finally, the ClePro web interface gives access to an interactive application called Popviz (https://popviz.vital-it.ch/#) that allows graphical visualization of the PTMs detected by our SLICE-SILAC approach.

### Non-lethal stress-induced proteolysis is CASP3/CASP7-dependent

With the aim of identifying proteins cleaved in a CASP3/CASP7-dependent manner in cells subjected to non-lethal stresses, a CASP3/CASP7 double knock-out (DKO) HCT116 cell line expressing VC3AI was generated (Supplementary Fig. S1B).

This DKO cell line was exposed to non-lethal stresses (2 μM cisplatin) and analyzed by SLICE-SILAC. Strikingly, none of the 92 proteins that were cleaved in VC3AI HCT116 exposed to such stress were found to be proteolytically processed in cells lacking CASP3 and CASP7 (Fig. 3C, Excel S4). This indicates that most, if not all, discrete protein cleavage induced by non-lethal stresses, require the activity of executioner caspases.

More than 90% of the proteins (393 out of 453) found to be cleaved in wild-type apoptotic cells (i.e. cells stimulated with 32 μM cisplatin) remained intact when CASP3 and CASP7 were invalidated (Fig. 3C, Excel S7). Hence, as expected, the majority of discrete proteolytic events occurring during apoptosis require the activity of executioner caspases. It is interesting to note that 29 proteins were still cleaved in cells lacking these caspases. This indicates that the proteases involved in such cleavage events are independently activated in cells undergoing death induced by high cisplatin concentrations. These proteases are thus not indirectly stimulated as a consequence of global CASP3/CASP7-mediated cell dismantling.

### Western blot validation of some SLICE-SILAC-detected cleavage events

**Fig. 4:**
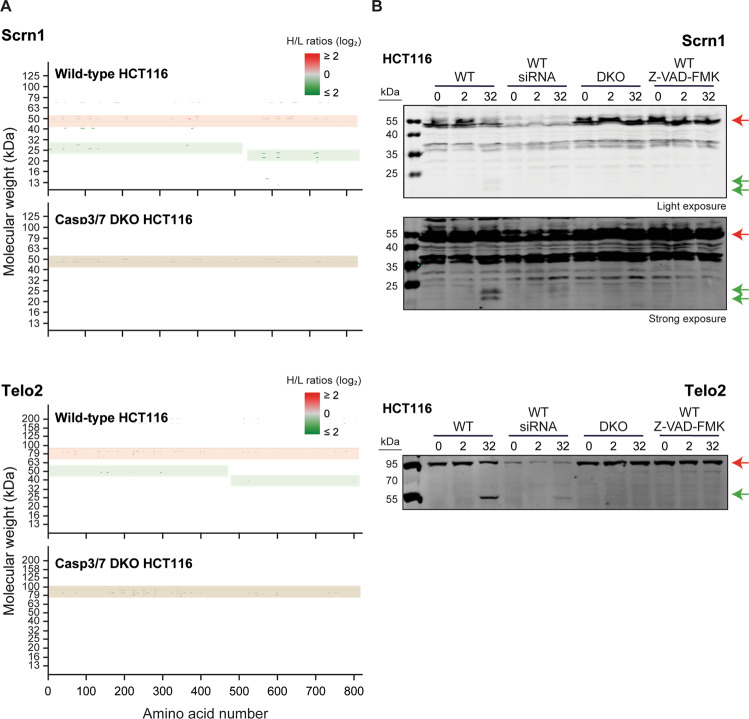
Validation of the CASP3/ CASP7-dependent cleavage of Scrn1 and Telo2 by Western blotting. **A** Visualization using the Popviz interface of the cleavage pattern of Scrn1 and Telo2 in HCT116 cells. **B** Wild-type and CASP3/CASP7 DKO HCT116 cells were incubated with the indicated cisplatin concentrations for 48 h and the presence of the indicated proteins was then evaluated by western blotting. The specificity of the antibodies used during the western blotting was assessed upon siRNA-mediated silencing of SCRN1 or Telo2 in cells (WT/siRNA lanes) prior exposure to cisplatin (see the methods). As an independent approach, caspases were inactivated by incubating the cells two hours prior cisplatin exposure with 50 µM of the pan caspase inhibitor Z-VAD-FMK, keeping the inhibitor in the culture medium when cisplatin was added. Red arrows point towards the full-length protein and green arrows to proteolytic fragments.

We attempted to validate, besides PARP1 (see Fig. 1D and in Excel S1–S4), the cleavage of several of the proteins identified by the SLICE-SILAC approach to be severed upon cisplatin exposure. We tested the specificity of 22 antibodies directed against 10 proteins by determining whether the band with the expected molecular weight for a given protein was decreased upon siRNA-mediated silencing (Table 2). Only two antibodies, those recognizing SCRN1 (Secernin-1) and Telo2 (Telomere length regulation protein TEL2 homolog) (Fig. 4B), were found to specifically recognize the expected proteins and at least one fragment on western blots.

**Table 2:** Antibodies used to validate the cleavage of proteins by western blotting.

Antibody against	Vendors	Catalog #	Silencing efficiency	Full-length detection	Fragment detection
Transportin-1	Antibodies-online	ABIN2838346	100%	No	No
Transportin-1	Abcam	ab163752	100%	Yes	No
Transportin-1	Abcam	ab191539	100%	No	No
TfR1	Cell Signaling Technology	13113 S	> 75%	Yes	Yes^a^
TfR1	Antibodies-online	ABIN2855434	> 75%	Yes	No
TfR1	Abcam	ab84036	>75%	Yes	Yes^a^
Multifunctional protein ADE2	Abcam	ab210806	Unresolved	Unresolved	No
Multifunctional protein ADE2	Abcam	ab171381	Unresolved	Unresolved	No
ATP citrate lyase	Abcam	ab117239	< 25%	No	No
ATP citrate lyase	Cell Signaling Technology	4332 S	< 25%	No	No
ATP citrate lyase	Abcam	ab226200	< 25%	Yes	No
TELO2	Abcam	ab122722	>75%	Yes	Yes
TELO2	Antibodies-online	ABIN2840034	> 75%	No	No
Secernin-1	Genetex	GTX123056	> 75%	Yes	No
Secernin-1	Genetex	GTX85172	> 75%	Yes	Yes
hFXR1p	Genetex	GTX33206	100%	Yes	Yes^a^
Plexin B2	Antibodies-online	ABIN525353	> 50%	No	No
Plexin B2	Abcam	ab193355	>50%	Yes	No
RP-A p70	Cell Signaling Technology	2193 S	> 75%	No	No
RP-A p70	Abcam	ab79398	>75%	Yes	No
RP-A p70	Cell Signaling Technology	2267 S	> 75%	Yes	No
p19A	Abcam	ab76502	>75%	Yes	No

This table indicates the proteins for which we tried to validate the cleavage observed in the Slice-SILAC experiments, and the antibodies we used for this purpose. The efficiency of the silencing is indicated as well if the antibody was able to detect the full-length protein and/or their fragment(s). “Unresolved” indicates that it was not possible to determine if the bands recognized by antibodies are specific for the protein of interest.

^a^Fragments also detected in non-treated cells.

## Discussion

In the present study, we show that CASP3 and/or CASP7 are mediating all the discrete proteolytic events that were detected in cells subjected to non-lethal stresses. This concern 92 proteins, 21 of which were unknown before to be cleaved in an executioner caspase-dependent manner (Excel S8). Some of these 21 proteins may play specific homeostatic functions to cope with the endured stress, such as the induction of anti-apoptotic responses as previously described in the context of the CASP3-mediated sequential cleavage of p120 RasGAP [7]. It may be that some of these proteins are innocent bystanders although we do not favor this possibility because of the very low executioner caspase activity found in cells subjected to non-lethal stresses, low activity that would not be expected to cause undue proteolytic events.

We cannot exclude that proteases other than CASP3 and CASP7 are activated by mild stresses but that the substrates they cleave are mostly of low copy number that would prevent their detection by our mass spectrometry analyses. Under the specific mild stress used here (low cisplatin concentration) and in the two cell lines tested (HCT116 and U2OS cells), we only evidenced the activity of executioner caspases. This may not be translatable to other cells and/or stress stimuli [66–68].

The majority of proteins cleaved in mild stress were also found to be cleaved during apoptosis, with the exceptions of Cullin-5 and CLIP-associating protein 1. Cullin-5 is part of the multiprotein Cullin-RING ligase 5 (CRL5) complex that targets a variety of substrates for proteosomal degradation [69, 70]. The cleavage of Cullin-5 under mild stress may hamper the formation of the complex, preventing the degradation of proteins that participate in cell survival. CLIP-associating protein 1 is kinetochore-associated protein required for the regulation of microtubule dynamics [71]. Its cleavage might prevent the development of mitosis until the damage in cells is resolved.

In some cases, we observed that the cleavage pattern of a given protein was different in cells exposed to non-lethal stresses and those undergoing apoptosis. One possibility is that PTMs other than proteolysis, such as phosphorylation that occurs during apoptosis [64], modulate the susceptibility of a protein to be cleaved by executioner caspases. It has been shown in this context that phosphorylation sites are seemingly enriched around caspase cleavage sites and that some protein cleavage can lead to phosphorylation site exposure [31], further affecting the migration of proteolytic fragments in gels. Another possibility, described for the p120 RasGAP protein [72], is the presence of 2 cleavage sites that display very different sensitivities to be recognized by caspases. In the presence of low caspase activities found in cells exposed to mild stresses, only the sensitive site is used, while both the sensitive and less sensitives sites are used during apoptosis. The differential cleavage of protein in cells expressing low or high caspase activity would consequently produce a different proteolytic pattern.

In summary, our work indicates that executioner caspases are activated in a homogenous manner in stressed non-dying cells and that these executioner caspases mediate all the cleavage events recorded in these cells (at least the cleavage of abundant proteins). We would predict that some of these cleavage events participate in adaptive homeostatic responses to cope with the stress the cells are exposed to, as shown earlier for the caspase-3-mediated p120 RasGAP sequential cleavage [7].

## Supplementary information

Supplementary information

Figure S1

Figure S2

Figure S3

Figure S4

Figure S5

Figure S6

Excel table S1

Excel table S2

Excel table S3

Excel table S4

Excel table S5

Excel table S6

table S7

Excel table S8

## Data Availability

Mass spectrometry proteomics data have been deposited in the PRIDE repository [73] from the ProteomeXchange Consortium [74] (dataset identifier PXD023488).

## Footnotes

1 The « 0 » in this section indicates the light isotope of the amino acids, while “+8” or “+10” correspond to the heavy isotope versions.
