# Supplementary material for: The proteolytic landscape of cells exposed to non-lethal stresses is shaped by executioner caspases
Source: Cell Death Discov. 2021 Jun 19;7:164. doi: 10.1038/s41420-021-00539-4 (PMC8257705; doi:10.1038/s41420-021-00539-4)
Supplement: Supplementary file 1 — Supplementary information [file 41420_2021_539_MOESM1_ESM.docx]

**Supplementary Table 1: Oligonucleotides used to generate the lentiviral vectors encoding the sgRNAs targeting caspase-3 and caspase 7**

| **Oligo name** | **Target gene** | **Exon** | **Sequence** |
| --- | --- | --- | --- |
| F.P.SGRNA32CAS3v2 | Caspase 3 | 2 | GGTATCCATGGAGAACACTG |
| R.P.SGRNA32CAS3v2 | Caspase 3 | 2 | AACCCATCTCAGGATAATCC |
| FP.SGRNA75CAS7 | Caspase 7 | 5 | GCTCCTAAGTATGCCAGGCA |
| RP.SGRNA75CAS7 | Caspase 7 | 5 | ATGCTTGGCAGACAATGGAC |

**Supplementary Table 2: Gating parameters for flow cytometry**

| **Name** | **Volts** | **Gain** |
| --- | --- | --- |
| FS | 388 | 1 |
| SS | 792 | 5 |
| FL1 (488 nm) | 260 | 1 |
| FL4 (675 nm) | 384 | 1 |

**Supplementary Table 3: Number of MS experiments per cell line and conditions**

| **Cell Line** | **Mild strees** | **Apoptosis** |
| --- | --- | --- |
| HCT116 | 3 | 3 |
| CASP3/CASP7 DKO HCT116 | 3 | 2 |
| U2OS | 3 | 2 |

**Supplementary Table 4: siRNA oligonucleotides**

|  | Oligo1 | Oligo2 |
| --- | --- | --- |
| Telo2 | 5'-CUUCUGCAGAGACUCAAGAdTdT-3' | 3'-dTdTGAAGACGUCUCUGAGUUCU-5' |
| SCRN1 | 5'-GUGCAGACUAUGAUGAACAdTdT-3' | 3'-dTdTCACGUCUGAUACUACUUGU-5' |

**Supplementary Table 5: Primers used for Casp3 KO validation TA cloning**

| Oligo name | Sequence |
| --- | --- |
| F.P.SGRNA32CAS3v2 | GGTATCCATGGAGAACACTG |
| R.P.SGRNA32CAS3v2 | AACCCATCTCAGGATAATCC |

**Legends to supplementary figures**

**Supplementary Figure S1: selection of HCT116 and U2OS clones expressing VC3AI and V3CAI expression in** **Casp3/ Casp7 DKO HCT116 cells**

Western blot of cell lysates from different HCT116 and U2OS clones isolated following infection with a lentivirus encoding the VC3AI CASP3/7 activity sensor (panel A) and from the CASP3/ CASP7 DKO HCT116 cell population infected with the same lentivirus (panel B). The antibody used in the western blot is an anti-GFP antibody that cross-reacts with VC3AI. GFP-expressing NB1 cells were used as control for the antibody and the respective parental cell lines as negative controls. HCT116 clone #5 and U2OS clone #12 (labelled in red in the figure) were those used in subsequent experiments.

Supplementary Figures S2 and S3: CASP3-like activity in HCT116 (Figure S2) and U2OS (Figure S3) cells in response to increasing doses of cisplatin

HCT116 and U2OS clones expressing the VC3AI CASP3-like activity sensor were seeded in 6 well plates (120’000 cells per well). The following day, cells were treated for 24, 48 or 72 hours with the indicated cisplatin concentrations. CASP3-like activity was assessed by flow cytometry.

Supplementary Figure S4: autofluorescence (in the GFP channel) of HCT116 and U2OS cells

Wild-type HCT116 and U2OS cells (120’000) were seeded in 6 well plates. The following day, cells were treated for 48 hours with increasing cisplatin concentrations. Cell-associated fluorescence was recorded by flow cytometry using laser settings to detect GFP in cells. The data correspond to the mean ± standard deviation of four independent experiments.

Supplementary Figure S5: Mass spectrometry identification capacity

The cellular proteome of U2OS [^43^](#_ENREF_43), consisting of 7287 proteins, was compared with the proteins that were detected in our experiments. The graphs depict, among low (less than 737 copies per cell), medium (738 to 11’389 copies per cell), and high (more than 11’389 copies per cell) abundance proteins, the distribution of those that were detected to have experienced PTMs (or not) and of those that were not detected by our SLICE-SILAC procedure in VC3AI HCT116 and VC3AI U2OS subjected to mild or apoptotic stresses.

**Supplementary Figure S6: Generation of CASP3/CASP7 DKO clones in HCT116 cells**.

The indicated clones were tested for the presence of CASP3 and CASP7 by western blotting. The CASP3 KO clone used to create the DKO cells was clone 33.2. See the methods for how these clones were generated using the CRISPR/Cas9 technology (A). DKO clones and wild type HCT116 cells (120’000 cells seeded in 2 ml of DMEM supplemented with 10% heat-inactivated FBS in wells of 6-well plates) were treated for the indicated times with FasL (50 ng/mL). The cleavage of PARP-1 was assessed in these clones (B). Clone H12 was used as the CASP3/CASP7 DKO cells in subsequent experiments.

**Legends to Excel Tables**

The following excel files summarize the results obtained with the SLICE-SILAC experiments and further analyzed using Popviz.

**Excel S1: Proteins found to have their gel migration pattern modified upon apoptosis induction**

VC3AI HCT116 and VC3AI U2OS cell lines, labeled with normal or heavy amino acid isotopes (120’000 cells seeded in wells of 6-well plates), were treated for 48 hours with 32 µM of cisplatin. Three different SLICE-SILAC experiments were performed for HCT116 cell line and two for U2OS. Proteins exhibiting the same profile in at least two experiments were considered for further analyses. Sheet “PTM proteins in apoptosis” displays all the proteins that presented changes in gel migration pattern after apoptosis triggering. Sheet “Cleavage” lists the cleaved proteins. Sheet “Tagging” describes proteins on which various (unidentified) chemical groups were added. Sheet “Degradation” lists proteins that were degraded after cisplatin exposure. Sheet “Ubiquitination”, lists ubiquitinated proteins. Sheet “Undefined” presents the proteins with unclear pattern of PTMs.

The file contains two sheets, “PTM – apoptosis” and “Cleavage details”. The first sheet lists the different PTMs observed in apoptotic cells and the second presents details about those proteins that are discretely cleaved.

Additional information on columns in the “cleavage details” sheet:

- Full-length protein molecular weight (based on migration in gel): full-length protein molecular weight (MW) based on migration in gel. These values were obtained via the Popviz interface, by pressing the Shift key on the keyboard and selecting at the same time the peptides that describe the full-length protein. The MW mean of the selected peptides is obtained.
- Cleavage: in which cell line was it observed and whether it was caspase-dependent or not. Additional information on the cleavage, when available, can also be found here.
- Cleavage sites: number of cleavage sites detected using the Popviz interface
- Detected fragments: number of fragments observed using the Popviz interface
- Molecular weight fragment 1, 2, 3, 4, 5: MW of the indicated fragments based on their migration in gels. These values can be obtained via the Popviz interface by pressing the Shift key on the keyboard and selecting the peptides of interest at the same time. The MW mean of the selected peptides is obtained.
- Fragment X MS coverage (X indicating which fragment is considered): for a given fragment, these columns list the region covered by the detected tryptic peptides during the MS analysis. The first number corresponds to the first amino acid of the first tryptic peptide found on the amino-terminal part of the fragment and the second number corresponds to the last amino acid of the last tryptic peptide found on the carboxy-terminal part of the fragment. Using the ClePro (Cleavage Profiling database) interface, one can hover on the purple line in the “cleavage pattern” box to highlight where this coverage lies in the sequence of the protein.
- Hypothetical fragment X boundaries (X indicating which fragment is considered): for a given fragment, these columns show where the fragment begins and ends in the sequence of the protein. This is inferred from the size of the proteolytic fragments detected via the MS analysis and the location of the most likely caspase cleavage site. Using ClePro, hovering on a given hypothetical fragment (blue line) in the “cleavage pattern” box will highlight where the fragment lies in the sequence of the protein.
- Region encompassing the first (or subsequent) cleavage site: these columns provide the region where a given cleavage site should be present (green lines in the “cleavage pattern” box of ClePro).

**Excel S2: Proteins found to have their gel migration pattern modified upon induction of non-lethal stress.**

VC3AI HCT116 and VC3AI U2OS cell lines, labeled with normal or heavy amino acid isotopes (120’000 cells seeded in wells of 6-well plates), were treated respectively, with 2 and 4 µM of cisplatin for 48 hours. Three different SLICE-SILAC experiments were performed for each cell line. Proteins exhibiting the same profile in at least two experiments were considered for further analyses. Sheets are organized and presented as in Excel S1.

**Excel S3: Cleaved proteins under mild stress or apoptotic that bear additional PTMs.**

This file describes the proteins found severed in VC3AI HCT116 and VC3AI U2OS cells when subjected to an apoptotic (sheet “Apoptosis”) or mild stress (sheet “Mild stress”) that display additional PTMs.

**Excel S4: Proteins cleaved in VC3AI HCT116 and VC3AI U2OS cell lines exposed to either non-lethal or apoptosis-inducing cisplatin concentrations**

This file indicates all the proteins cut in VC3AI HCT116 and VC3AI U2OS cell lines when exposed to an apoptotic (sheet “Cleaved during apoptosis”) or mild stress (sheet “Cleaved during mild stress”). In both sheets is indicated the protein names, their accession number and a description of the cleavage pattern (column Pattern description) that tells in which cell line the cut was observed.

**Excel S5: Previously undescribed protease substrates.**

Proteins found cleaved in apoptosis, in VC3AI HCT116 and VC3AI U2OS cell lines (Excel S4), that were not described as cleaved in the Degrabase database, are listed here. Accession number and Protein names are indicated.

**Excel S6: Proteins cleaved under mild stress conditions exhibiting a different cleavage pattern in apoptosis**

The patterns of the proteins found cleaved in VC3AI HCT116 and VC3AI U2OS cell lines, when exposed to mild stress, were compared with the ones exhibited when the same cells were subjected to the apoptotic stress. Proteins exhibiting a different cleaved pattern between these conditions are listed here. Their protein name and accession number are shown, as well the number of cleavage sites and fragments detected for each of these proteins in apoptosis and mild stress.

**Excel S7: Proteins cleaved in account of Casp3/ Casp7 in apoptosis.**

Casp3/ Casp7 DKO VC3AI HCT116 cell, labeled with normal or heavy amino acid isotopes (120’000 cells seeded in wells of 6-well plates), were treated for 48 hours with 32 µM of cisplatin. Three different SLICE-SILAC experiments were performed. Proteins exhibiting the same profile in at least two experiments, were compared to the list of proteins found severed in VC3AI HCT116 cells subjected to the apoptotic stress. Proteins not longer appearing as cleaved in the CASP3/CASP7 DKO VC3AI HCT116 cells were considered severed in a Casp3 /Casp7- dependent manner. Sheet “Casp3-7 independent cleavage” shows proteins cleaved by other protease different to Casp3/Casp7 and sheet “Casp3-7-dependent cleavage” indicates the proteins cleaved in account of Casp3/ Casp7. In both sheets is indicated the protein names, their accession number and a description of the cleavage pattern (column Pattern description) that tells in which cell line the cut was observed.

**Excel S8: Proteins cleaved in mild stress previously unknown to be cleaved.**

This file lists the proteins found cleaved when VC3AI HCT116 and VC3AI U2OS cell line were exposed to a mild stress (2 and 4 µM of cisplatin respectively, Excel S3) and were previously unknown to be severed in any condition. Accession number and Protein names are indicated.
