# Supplementary figures and images for: The proteolytic landscape of cells exposed to non-lethal stresses is shaped by executioner caspases
Source: Cell Death Discov. 2021 Jun 19;7:164. doi: 10.1038/s41420-021-00539-4 (PMC8257705; doi:10.1038/s41420-021-00539-4)

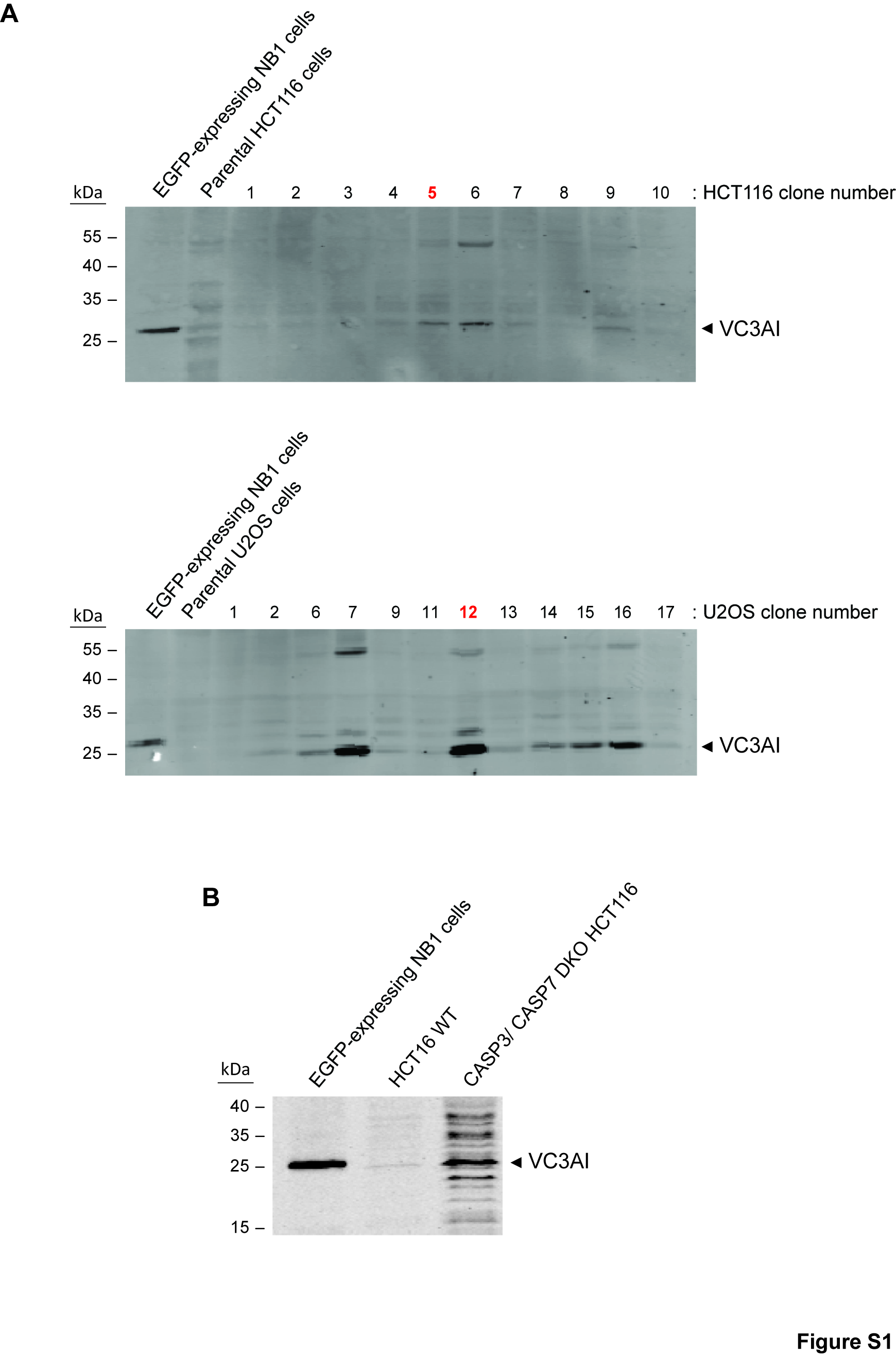

Supplement: Supplementary file 2 — Figure S1 [file 41420_2021_539_MOESM2_ESM.tif]

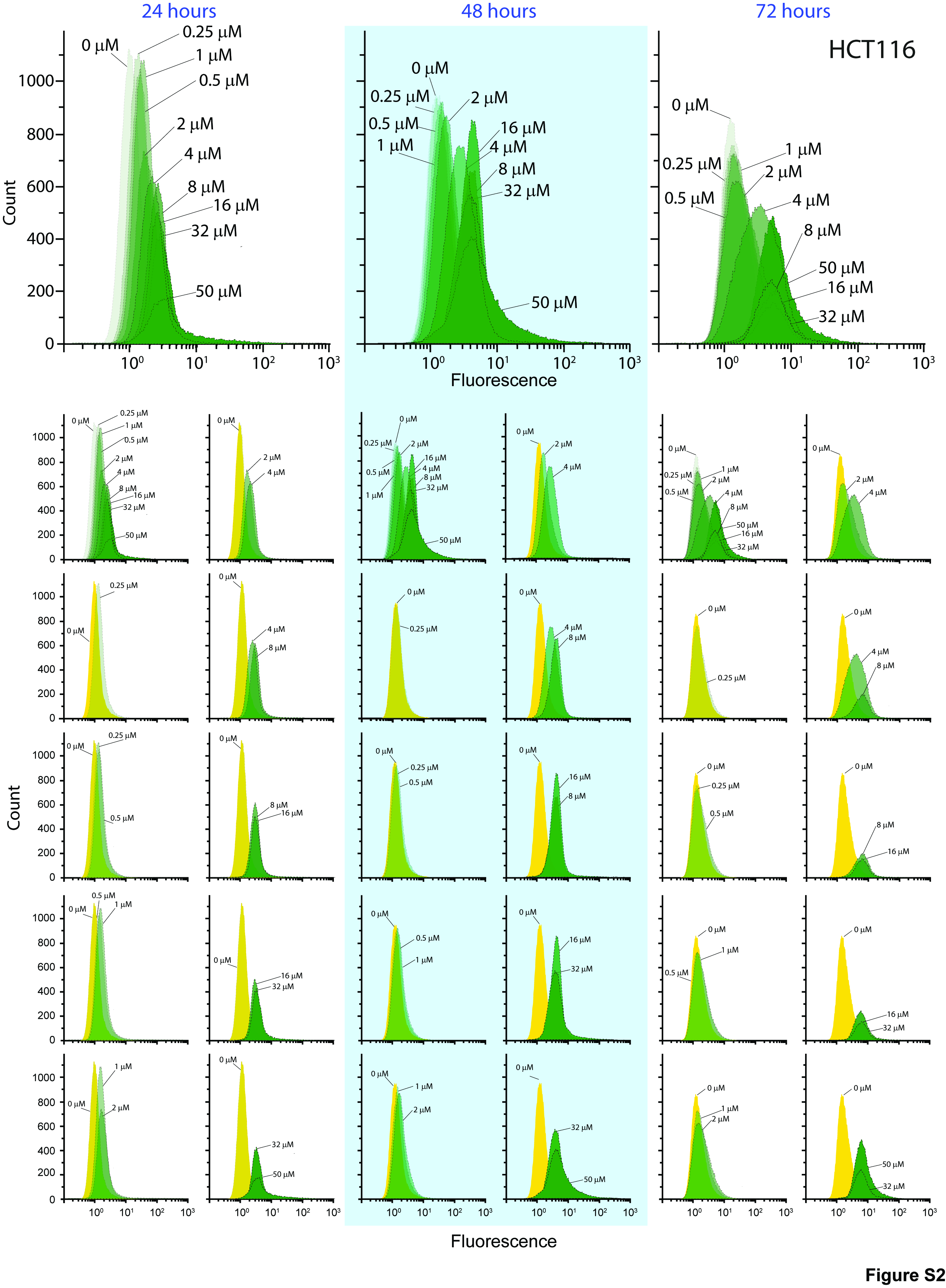

Supplement: Supplementary file 3 — Figure S2 [file 41420_2021_539_MOESM3_ESM.tif]

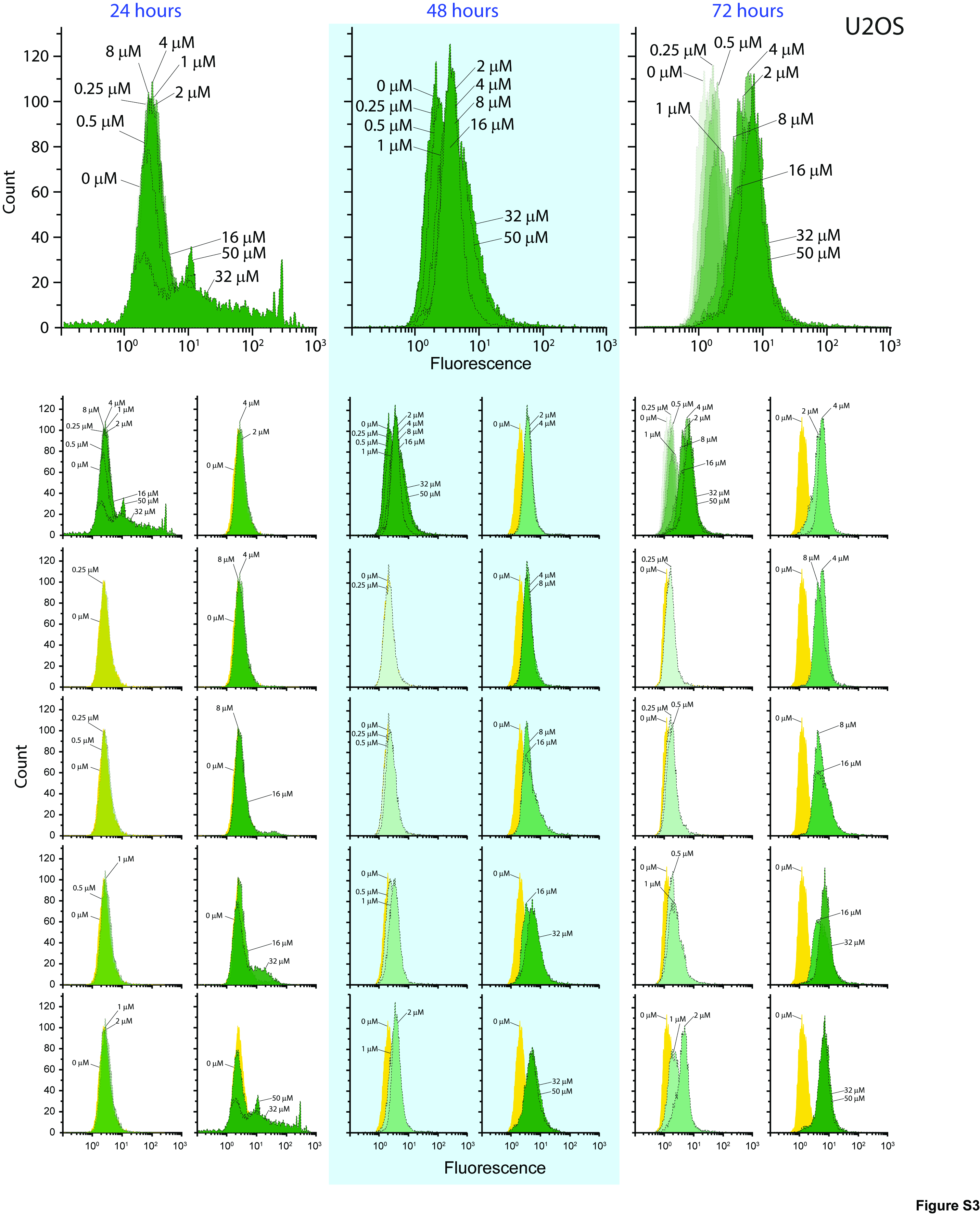

Supplement: Supplementary file 4 — Figure S3 [file 41420_2021_539_MOESM4_ESM.tif]

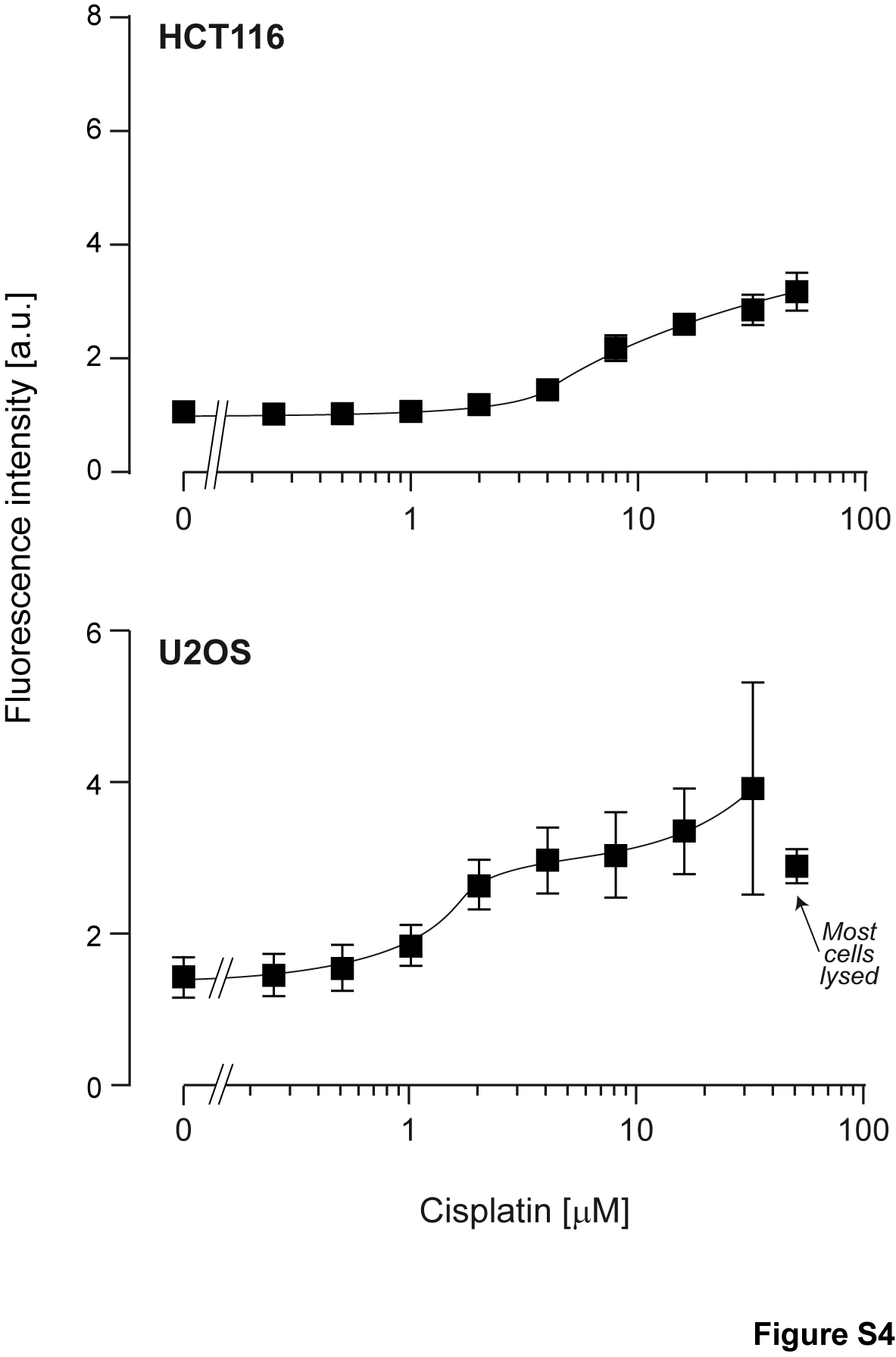

Supplement: Supplementary file 5 — Figure S4 [file 41420_2021_539_MOESM5_ESM.tif]

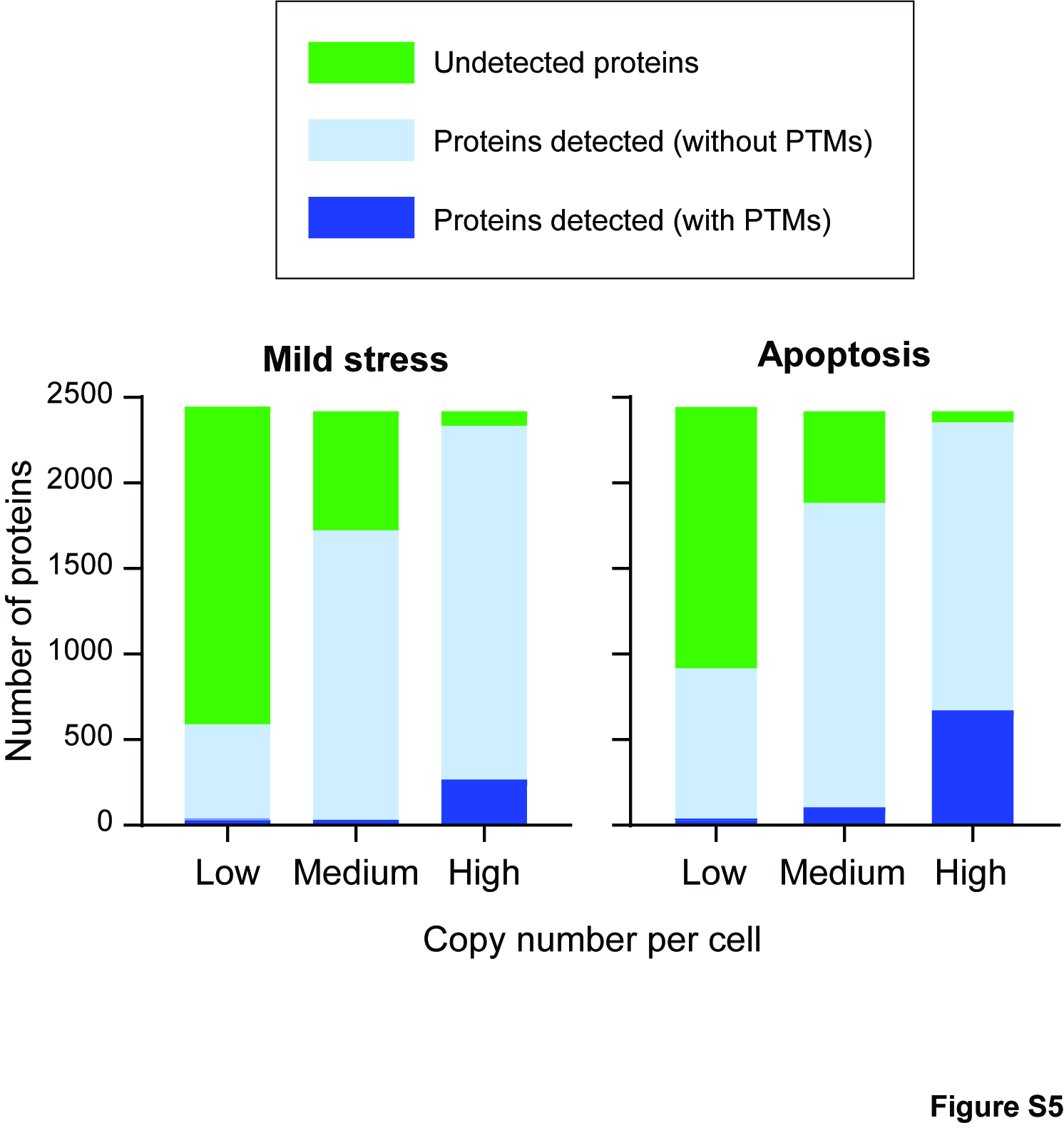

Supplement: Supplementary file 6 — Figure S5 [file 41420_2021_539_MOESM6_ESM.tif]

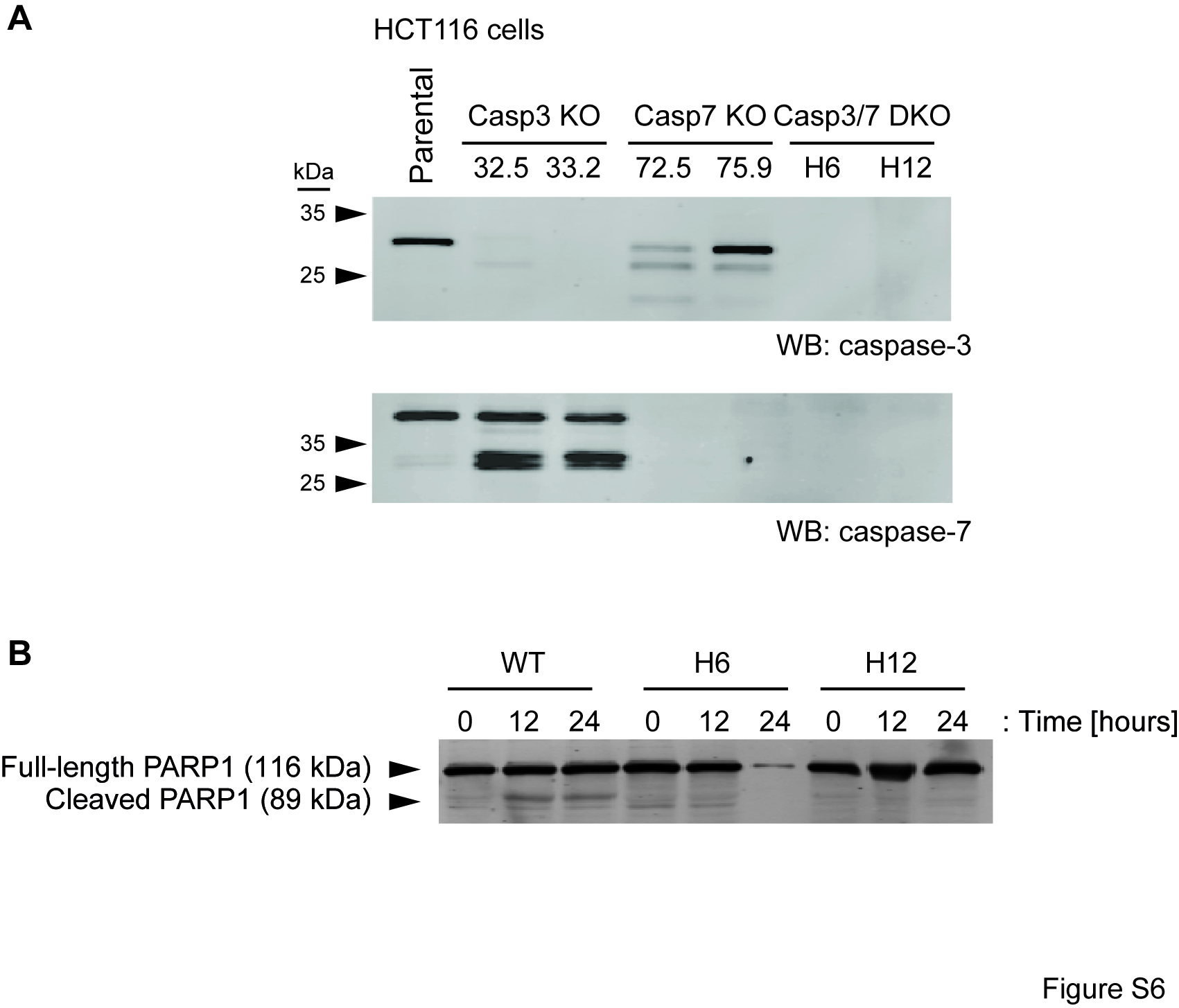

Supplement: Supplementary file 7 — Figure S6 [file 41420_2021_539_MOESM7_ESM.tif]
